# Significant decrease of adenocarcinoma *in situ* not reflected in cervical adenocarcinoma incidence in the Netherlands 1989–2003

**DOI:** 10.1038/sj.bjc.6604118

**Published:** 2008-01-08

**Authors:** H P van de Nieuwenhof, L F A G Massuger, J A de Hullu, M A P C van Ham, J A A M van Dijck, A G Siebers, R L M Bekkers

**Affiliations:** 1 Department of Gynaecology/Obstetrics, Radboud University Nijmegen Medical Centre, Nijmegen, The Netherlands; 2 Netherlands Cancer Registry, Association of Comprehensive Cancer Centres, Utrecht, The Netherlands; 3 Department of Epidemiology and Biostatistics, Radboud University Nijmegen Medical Centre, Nijmegen, The Netherlands; 4 Department of Pathology, Radboud University Nijmegen Medical Centre, Nijmegen, The Netherlands

**Keywords:** adenocarcinoma, adenocarcinoma *in situ*, incidence

## Abstract

Over the period 1989–2003, the incidence of cervical adenocarcinoma (*n*=1615) was stable whereas that of cervical adenocarcinoma *in situ* (*n*=1884) significantly decreased (*P*=0.008), mainly caused by adenocarcinoma *in situ* lesions with a concurrent squamous dysplasia.

Adenocarcinoma (AC) and adenocarcinoma *in situ* (ACIS) of the uterine cervix are rare diseases with an incidence of 1.2 per 100 000 women per year for AC (http://www.oncoline.nl). The incidences of both have increased during the last three decades, especially in women under the age of 40 years (Zheng *et al*, 1996; Vizcaino *et al*, 1998; Bergstrom *et al*, 1999; Plaxe and Saltzstein, 1999; Smith *et al*, 2000; Liu *et al*, 2001; Sasieni and Adams, 2001; Visioli *et al*, 2004; Wang *et al*, 2004; Bray *et al*, 2005). This contrasts with the decrease in squamous cell carcinoma (SCC) since the introduction of screening programmes (Bergstrom *et al*, 1999; Ronco *et al*, 2005; Kyndi *et al*, 2006). Studies have shown that Pap smears are less sensitive in detecting precursor lesions of AC than those of SCC (Etherington and Luesley, 2001; Schoolland *et al*, 2002; Kalir *et al*, 2005). This makes the incidence of AC less likely to be influenced by screening (Nieminen *et al*, 1995; Bergstrom *et al*, 1999).

Adenocarcinoma *in situ* is generally considered a precursor lesion for AC (Kurian and al-Nafussi, 1999; Zaino, 2002), but whether glandular precursor lesions develop in a manner similar to squamous lesions, that is from mild via moderate to severe atypia, is still under discussion (Brown and Wells, 1986; Hitchcock *et al*, 1993; Goldstein *et al*, 1998).

We examined the incidence of AC and ACIS to see whether a change in AC incidence is preceded by a similar change in ACIS, especially in young women.

## PATIENTS AND METHODS

Data concerning 2075 cases of ACIS/severe atypia registered between 1 January 1992 and 31 December 2003 in the Netherlands were collected via PALGA, the nationwide Netherlands database of histo- and cytopathology (Casparie *et al*, 2007). This database had national coverage from 1992. In total, 191 individuals were excluded: 96 individuals who developed an adenocarcinoma of cervical, endometrial, ovarian, or tubal origin within 6 months of ACIS diagnosis, and another 95 individuals with the cytological diagnosis of ACIS/severe glandular atypia not confirmed histologically. A total of 1884 women were included in the study.

Data concerning the incidence of AC were obtained from the nationwide Netherlands Cancer Registry (Association of Comprehensive Cancer Centres), which was set up in 1989, and cases of AC during the years 1989–2003 were retrieved (1615 patients). The population data for the Netherlands for calculating European standardised rates (ESRs) were obtained from the database of Statistics Netherlands (http://www.cbs.nl/).

The incidence of AC and that of ACIS were calculated per 100 000 person years (ESR). Age was categorised into 15-year strata as follows: 15–29, 30–44, 45–59 and >60 years. To avoid small numbers, 3-year groupings of the years from 1989 to 2003 were used. Trends in incidence rates were examined by calculating the estimated annual per cent change (EAPC). Using a univariate general linear model, statistical differences between the incidences over time were calculated. A significance level of *P*<0.05 was chosen.

## RESULTS

The mean age of patients with ACIS was 37.8 years (range 17–93): 36.7 (s.d. 8.39) years for patients with a concurrent squamous lesion, compared to 39.3 (s.d. 9.75) years for patients without a concurrent squamous lesion (*P*<0.001). In patients with ACIS, 1078 patients (57%) had a concurrent squamous dysplasia: 92 patients had atypical squamous cells, 112 patients had mild dysplasia, 152 patients had moderate dysplasia, 421 patients had severe dysplasia and 301 patients had squamous cell carcinoma *in situ*.

**Figure 1:**
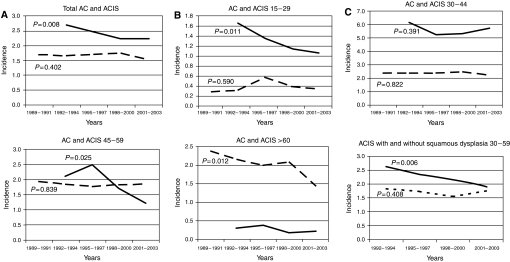
(**A**) Total age standardised incidences for ACIS (1992–2003) and AC (1989–2003). (**B**–**E**) Age standardised incidence for ACIS (1992–2003) and AC (1989–2003) for patients of different years of age. (**F**) Age standardised incidence for ACIS, subdivided between ACIS with and without a concurrent squamous dysplasia. (**A**–**E**) ACIS incidence is indicated with a bold line, AC incidence with a dotted line. (**F**) ACIS with concurrent squamous dysplasia is indicated with a bold line, without squamous dysplasia with the dotted line.

Figure 1A shows the overall incidence of AC and ACIS over time. The overall incidence of AC remained unchanged (*P*=0.402), whereas the overall incidence of ACIS decreased significantly (*P*=0.008). In Figure 1B–E, the age-standardised incidences of AC and ACIS are shown for different age groups. A significant decrease in ACIS incidence was found for the age groups 15–29 and 45–59. A significant decrease in AC incidence was found for the age groups over 60 years of age. No significant increase was found in AC incidence in women aged 15–29 years. In Figure 1F, the incidence of ACIS subdivided between patients with and without concurrent squamous dysplasia is shown for patients eligible for the screening programme (30–59 years of age). In the group of patients with a squamous dysplasia, a significant decrease is seen (*P*=0.006), whereas in the patients without concurrent squamous dysplasia, the decrease is not significant (*P*=0408).

## DISCUSSION

This nationwide study in the Netherlands shows a significant decrease in the incidence of ACIS but an unchanged incidence of AC. Moreover, the decrease in the incidence of ACIS is mainly caused by ACIS lesions with a concurrent squamous dysplasia. The only two previous studies of AC and ACIS trends, both in the United States, found an increase in the incidence of ACIS without a significant change in AC incidence over a 20-year period (Plaxe and Saltzstein, 1999; Wang *et al*, 2004).

This stable pattern in AC incidence accords with an earlier report from the Netherlands (Bulk *et al*, 2005). However, in contrast to that study, no significant increase was seen in AC incidence in young patients. We used the same database but extended the analysis to an additional 5 years, making short-term fluctuations in AC incidence less important.

This unchanged AC incidence contrasts with the incidence rates reported from other countries where it is rising (Zheng *et al*, 1996; Vizcaino *et al*, 1998; Bergstrom *et al*, 1999; Plaxe and Saltzstein, 1999; Smith *et al*, 2000; Liu *et al*, 2001; Sasieni and Adams, 2001; Visioli *et al*, 2004; Wang *et al*, 2004; Bray *et al*, 2005). However, a stable pattern has been reported more often (Vizcaino *et al*, 1998; Chan *et al*, 2003; Bulk *et al*, 2005), although a decreasing incidence has also been recorded (Vizcaino *et al*, 1998; Bray *et al*, 2005; Howlett *et al*, 2007). Studies covering the years after 1995 more often found a decrease in AC incidence after an earlier increase; for example, Howlett *et al* (2007) found an increase in AC incidence from 1981 to 1995 and a decrease in incidence from 1995 to 2001. This was attributed to the change in the sampling device used; the endocervical brush was introduced to replace the spatula that focused only on the sampling of squamous cells. In a study of the screening history and risk of AC, it was concluded that AC incidence should begin to decline in the current decade among screened women (Mitchell *et al*, 2003). Our study included data from 1989 to 2003, the second study to incorporate years after 2000 (Howlett *et al*, 2007).

The significant decrease of ACIS incidence in this study contrasts with two other studies describing an increase, mainly in patients under age 40 years (Plaxe and Saltzstein, 1999; Wang *et al*, 2004). In the light of the increasing prevalence of HPV and sexually transmitted infections in young women due to younger ages of first intercourse (http://www.rivm.nl), the decrease in ACIS incidence is rather surprising and contrasts with the increasing incidence of other HPV-related lesions like vulvar intraepithelial neoplasia (Jones *et al*, 1997; Iversen and Tretli, 1998; Joura *et al*, 2000). The decrease in ACIS incidence was mainly caused by ACIS lesions with a concurrent squamous dysplasia. In the last few years, much attention has been paid to the detection and treatment of CIN lesions in the prevention of SCC. Treatment of these patients with CIN may have caused removal or prevention of the ACIS lesion. The significantly younger age of patients with ACIS and concurrent squamous dysplasia could mean that lesions with concurrent squamous dysplasia are detected in an earlier phase than solitary ACIS lesions are. Alternatively, the difference in ACIS with and without a squamous dysplasia may reflect a decline over time in the level of detail of histopathological reporting. In this study, 57% of ACIS lesions had a concurrent squamous dysplasia, a proportion according with other studies (Colgan and Lickrish, 1990; Anciaux *et al*, 1997; Casper *et al*, 1997; Bekkers *et al*, 2003).

The decrease in ACIS incidence was mostly seen in the 15–29 and 45–59 years age groups, the former outside the ages of 30–60 years in the screening programme in the Netherlands. The decrease may be the result of a general decrease in opportunistic screening in this age group. At ages 30–44, when no significant decrease in ACIS was seen, the first cervical smear in the screening programme is performed. Because of the first screening effect, the incidence in this age group is not likely to decrease. The women aged 45–59 probably had an earlier cervical smear, which may have led to an ACIS decrease.

Others report that AC incidence might not be influenced by screening (Nieminen *et al*, 1995; Bergstrom *et al*, 1999). The decrease of ACIS in the age group 45–59 and of AC over age 60 years in this study may reflect detection of ACIS lesions in the screened population, but further studies will be required over long time periods.

The present study finds a decrease in ACIS incidence over time, mainly due to lesions with a concurrent squamous dysplasia, but not (yet) reflected by a decrease in AC incidence.
